# Hybrid fractal acoustic metamaterials for low-frequency sound absorber based on cross mixed micro-perforated panel mounted over the fractals structure cavity

**DOI:** 10.1038/s41598-022-24621-8

**Published:** 2022-11-28

**Authors:** Sanjeet Kumar Singh, Om Prakash, Shantanu Bhattacharya

**Affiliations:** 1 Department of Design, Indian Institute of Technology Kanpur, Kanpur, Uttar Pradesh 208016 India; 2 Microsystem Fabrication Laboratory, Department of Mechanical Engineering, Indian Institute of Technology Kanpur, Kanpur, Uttar Pradesh 208016 India; 3 Boeing International Corporation India Private Limited, RMZ Infinity, Tower D, 5th Floor, Old Madras Road, Bengaluru, Karnataka 560001 India

**Keywords:** Applied physics, Acoustics, Mechanical engineering

## Abstract

The proposed work enumerates a hybrid thin, deep-subwavelength (2 cm) acoustic metamaterials acting as a completely new type of sound absorber, showing multiple broadband sound absorption effects. Based on the fractal distribution of Helmholtz resonator (HRs) structures, integrated with careful design and construct hybrid cross micro-perforated panel (CMPP) that demonstrate broad banding approximately one-octave low-frequency sound absorption behavior. To determine the sound absorption coefficient of this novel type of metamaterial, the equivalent impedance model for the fractal cavity and the micro-perforated Maa’s model for CMPP are both used. We validate these novel material designs through numerical, theoretical, and experimental data. It is demonstrated that the material design possesses superior sound absorption which is primarily due to the frictional losses of the structure imposed on acoustic wave energy. The peaks of different sound absorption phenomena show tunability by adjusting the geometric parameters of the fractal structures like cavity thickness ‘*t*’, cross perforation diameter of micro perforated panel, etc. The fractal structures and their perforation panel are optimized dimensionally for maximum broadband sound absorption which is estimated numerically. This new kind of fractals cavity integrated with CMPP acoustic metamaterial has many applications as in multiple functional materials with broad-band absorption behavior etc.

## Introduction

Numerous uses for the deep-subwavelength thick broadband low-frequency sound absorber can be found in acoustic cloaking and noise reduction. An acoustic metamaterial is an excellent candidate to tackle all challenges with careful design of structures that may possess extraordinary acoustic properties like broadband noise absorption^1–5^, sound insulation^6–8^, noise cloaking properties^9,10^, acoustic jetting properties^11^ etc. Acoustic metamaterials are well known as artificial or man-made structures that may be programmed through negative effective density^12,13^, negative effective modulus^14,15^, and simultaneous negative modulus and density^16–18^. Researchers have recently proposed 2D fractals acoustic metamaterials^19^ and 3D labyrinthine fractal acoustic metamaterials^20^, which can possess multi-band sound blocking properties in the low frequency domain. Another broadband low frequency sound isolator is designed through a spider web-inspired membrane-type meta-material^21^. Researchers may seek to find the lightweight structures of different material designs to possess excellent sound absorption to solve challenges related to noise control^22,23^. Further, it has long been a challenge to get broadband sound absorption while keeping thin and light weightedness as structural properties. Metamaterial design like multi-coiled structures^24^, can achieve perfect absorption at extreme low frequency of 50 Hz with a thickness of 1.3 but cannot tune once it is fabricated. Researchers have also tried conventional micro-perforated panels (MPP)^25–28^, with back cavity, Cascade neck-embedded Helmholtz resonators based metamaterials^29^, MPP with neck-embedded Helmholtz resonator^30^, and successfully achieved an overall good sound absorption level at low frequencies. However, the thickness of the backing cavity is usually more than 5 cm for obtaining a broadband sound absorption behavior. Ultrathin membrane metamaterials (MM)^31,32^, are a very good candidate for broadband sound absorption behavior but the problem in MM is membrane loosening effect which may come in due course of time after repeated use.

**Figure 1:**
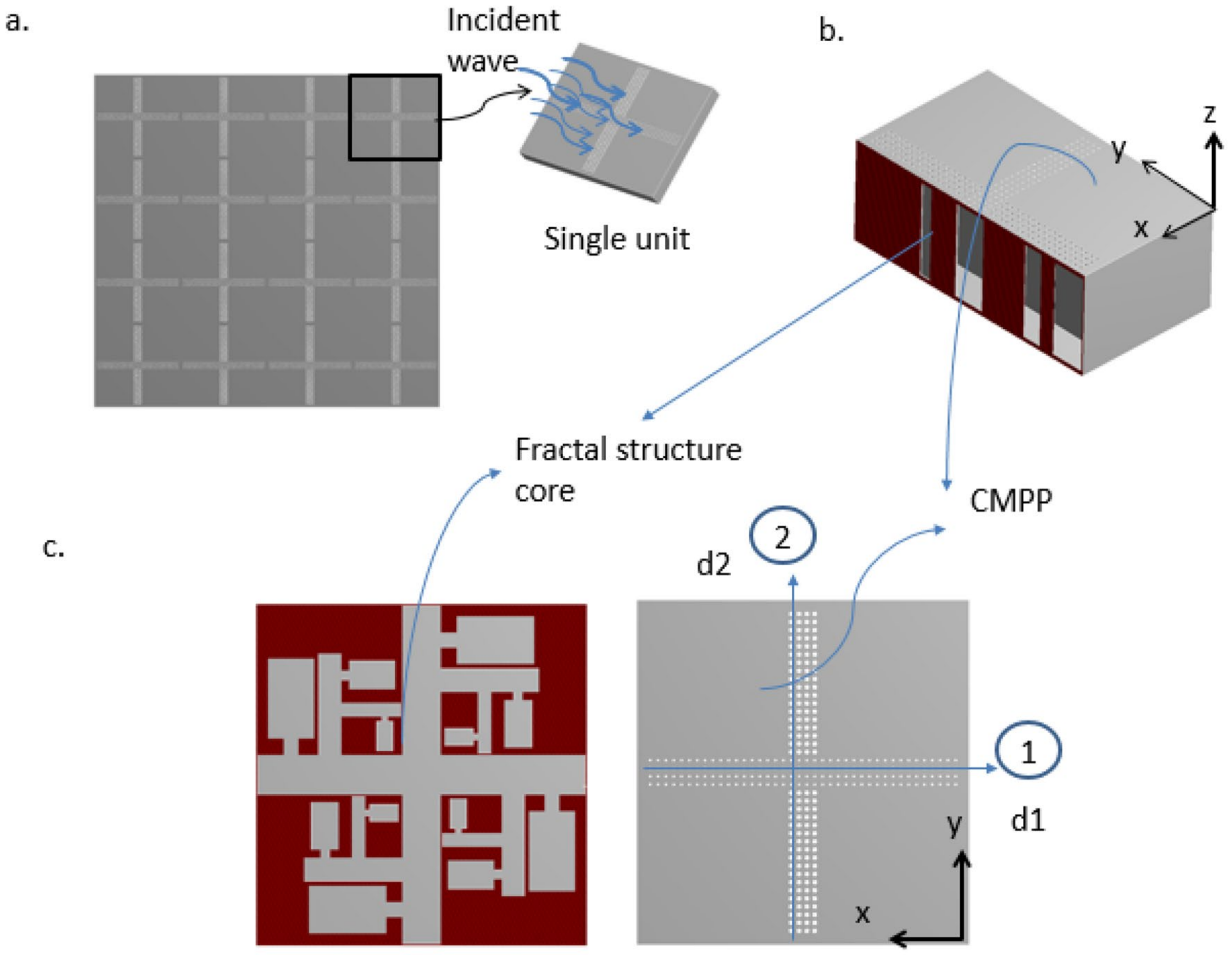
(**a**) Schematic of cross perforated fractal structure hybrid metamaterials panel composed of a cross-micro-perforated top face-sheet, a Helmholtz swastika fractals structure as core, and a back plate as bottom face-sheet. (**b**) One unit cell, sidewall cut-off vertically to see details inside. (**c**) CMPP with different perforation sizes in direction one having perforation diameter d1 and direction two is d2 and the back fractals cavity. Courtesy (ANSYS 17.0^45^).

This article has developed a new type of tunable micro-perforated face-sheet design (with perforation diameter ≤ 1 mm) backed up by fractal geometry as shown in Fig. 1, of subwavelength dimensions that demonstrate excellent broadband sound absorption behavior. The thickness of this classical metamaterial design is less than 2 cm, and it can be easily programmed/tuned according to the industrial need and scope in different fields.

The series–parallel circuit analogy is applied to obtain an equivalent impedance method through which a theory is proposed, to calculate the sound absorption coefficient is established for this new class of fractal designs. This works also experimentally validates and compares with the theoretical model and a finite element model. Perfect sound absorption is achieved around 1000 Hz, together with broadband sound absorption starting from 400 to 1600 Hz, when the thickness of these unique metamaterials is about 20 mm. Almost perfect sound absorption has been found around 1000 Hz, together with one-octave relative absorption bandwidth starting from 600 Hz, when the thickness of the metamaterials is just 20 mm with the integration of two-unit cells. We have also integrated the four-unit cells to achieve broadband sound absorption and successfully achieved 61% higher relative bandwidth and sound absorption coefficient greater than 80%.

## Theoretical model

The proposed acoustic metamaterial having two face-sheet and a swastika fractal structure core, as shown in Fig. 1. The cad model of the proposed metastructre is design in Design Modeler of ANSYS 17.0^45^. The top face-sheet consist of cross perforation having uniform perforation diameter in direction ‘1’ (d_1_) and direction ‘2’ as (d_2_), in a micro-perforated panel (CMPP). The unique Helmholtz resonators are fractally distributed and act as an acoustic cavity core with the bottom face-sheet in the shape of a rigid backing plate. The fractal core is designed based on novel fractal shapes and contains multiple Helmholtz resonators along the arms of the fractal shape. In the first level branches, the unit shape (side branched Helmholtz resonator) is rotated by $90^\circ ,180^\circ \, \mathrm{and} \, 270^\circ$ respectively to create four arms of the fractal structure and interconnected in a crossroad like manner. As the Helmholtz arm iterates ‘n’ times to a scaled down geometry connected to the base arm (Helmholtz resonators) at every 90°. Rotation angle a scale factor of 0.6 is used for every iteration. The final acoustic meta-structure up to which we investigate corresponds to an “n” value of 3 and the final core structure shown in Fig. 1c.

In general, the absorption coefficient of any acoustic metamaterials with a rigidly backed panel can be estimated through its impedance as

1 $$\propto =1-{\left|\frac{{z}_{s}/{z}_{0}-1}{{z}_{s}/{z}_{0}+1}\right|}^{2},$$

where, ${z}_{s}$ is known as the surface impedance of the acoustic absorber. ${z}_{0}={\rho }_{0}{c}_{0}$ is the characteristic impedance where ${\rho }_{0}$ and ${c}_{0}$ are mass density and sound speed in air, respectively. The surface impedance of the proposed fractal CMPP have been calculated as:

2 $${{z}_{s }=z}_{Mp}+{z}_{fc},$$

where, ${z}_{Mp}$ and ${z}_{fc}$ are the acoustic impedance of the CMPP and the fractal structure containing cavity, respectively.

The impedance of the side branch resonator Zr at X_1_ as shown in Fig. 1b in Supplementary Materials, is expressed as^33–35^

3 $${\text{Zr }} = \, - {\text{jZ}}_{{{\text{c}} }} {\text{cot}}\left( {{\text{kh}}} \right) \, + {\text{ Z}}_{{\text{h}}} ,$$

4 $${\mathrm{Z}}_{\mathrm{h}} = \frac{\rho c}{{s}_{h}} \left[ 0.0072+jk \left(l+0.75\right)\right],$$

where Zc = $\rho c/{s}_{c}$ represents the impedance of the helmholtz cavity and Z_h_ represents the neck impedance of the resonators as suggested by Seo et al.^33^. $l$ is the neck length, ${s}_{c}$ is the cross sectional area of the cavity, $h$ is the height of the cavity. here $k=2\pi f/c$ is the wave number.

‘S_h_’ is the cross- sectional area of the neck region. We first calculated the equivalent impedance of the fractals cavity ${z}_{fc}$ through an electrical analogy (see Supplementary Material for more details).

Impedance of the micro-perforated plate can be calculated by Maa’s Model^36,37^

5 $${\mathrm{MPP} \, \mathrm{impedance } \quad z}_{m}=r+j\omega m,$$

where

6 $$r=\frac{32\eta }{\varnothing {\rho }_{0}{c}_{0}}\frac{t}{{d}^{2}} \left(\sqrt{1+\frac{{x}^{2}}{32}} +\alpha \frac{\sqrt{2}}{8} x \frac{d}{t}\right),$$

and

7 $$m=\frac{t}{\varnothing {c}_{0}} \left(1+\frac{1}{\sqrt{9+\frac{{x}^{2}}{2}}}+0.85 \alpha \frac{d}{t}\right),$$

where $\varnothing =$ Porosity, $\eta$ = dynamic viscosity, $\alpha$ = perforation constant, d = diameter of hole, t = thickness of the perforated plate, $x=d\sqrt{\frac{\omega {\rho }_{0}}{4 \eta }}.$

We can now calculate the total impedance of the cross perforated plate of single unit as

8 $${z}_{Mp}={\left(\frac{1}{zm1}+\frac{1}{zm2}\right)}^{-1},$$

where $zm1$ is the impedance of the CMPP in direction 1 and $zm2$ is the impedance of the perforation in direction 2 as shown in Fig. 1c.

We are thus enabled to put the Eqs. (8) and (13) (Supplementary Material) as inputs to the Eq. (2) and we can thereby theoretically calculate the sound absorption coefficient of the cross micro-perforated fractal acoustic metamaterials with the help of Eq. (1). The absorption coefficient spectrum of the theoretical model is obtained by using MATLAB (R2016a)^46^.

**Figure 2:**
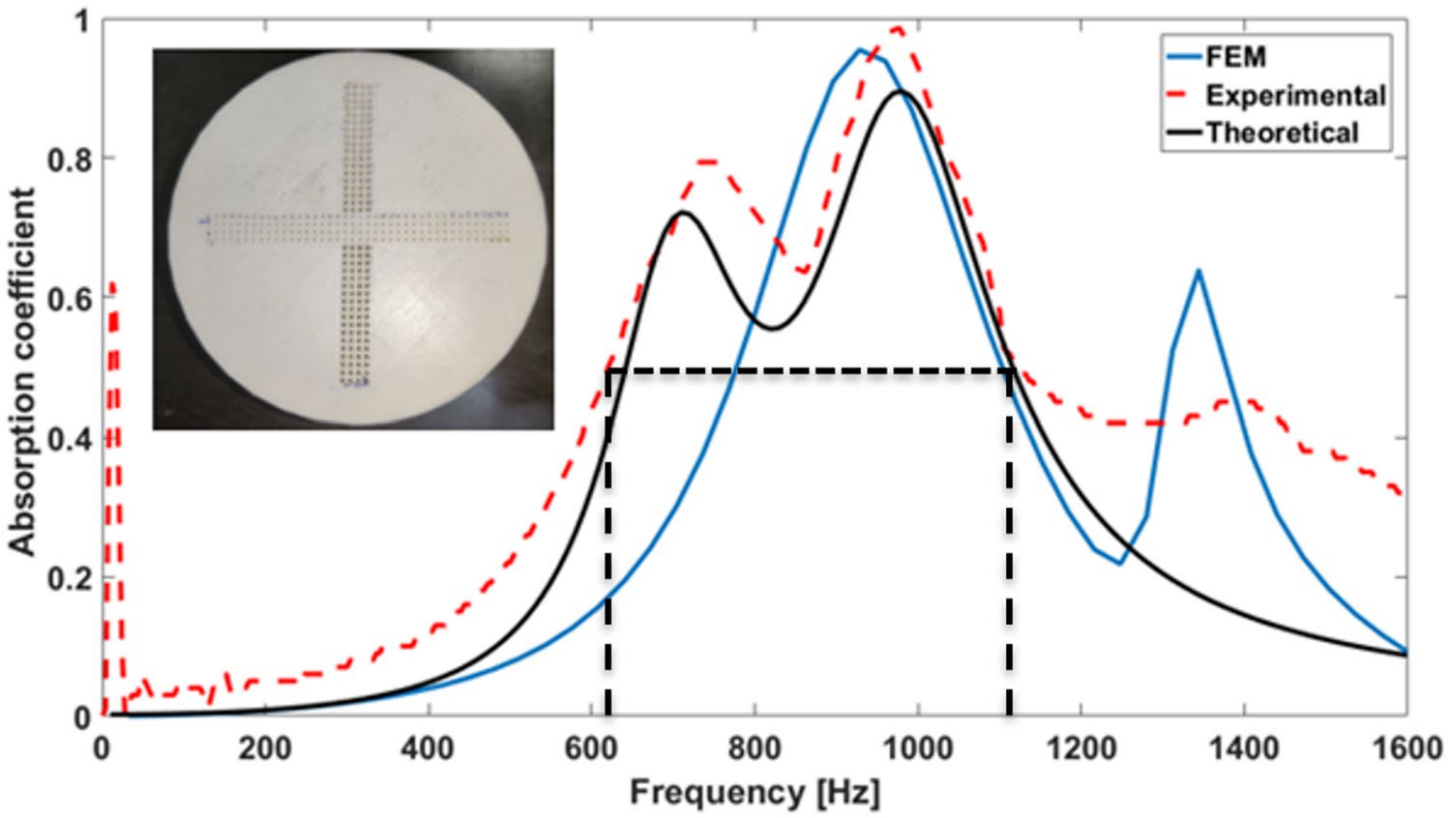
Sound absorption coefficient of CMPP predicted by the analytical method, FEM model and experimental results. Courtesy (MATLAB R2016a)^46^.

Figure 2 depicts the broadband sound absorption in this case. As we can observe clearly that the initial peak of the absorption coefficient of the experimental result is not seen in the FEM simulation values, and similarly the last peak is not seen in the theoretical model. Although the experimental, theoretical, and numerical results have good consistency in terms of the amplitude at maximum absorption etc., in theoretical models, the linear superposition principle has been used as shown in Eq. (8). Thus, the nonlinear coupling effect of the perforation and cavity of the two distinctly truncated acoustic signals passing from the two different diameter holes on the cover plate is not considered while determining the theoretical spectrum. In numerical simulations, the CMPP has been assumed to be equivalent to a porous rigid body, and the visco-thermal losses across the fractal core has been considered to be negligiblemal^38–41^. Thus the first absorption peak as is distinctly observed in experimental data as well as theoretical prediction does not show up in the simulation data. It also observed that the experimental frequency bandwidth is wider than numerical and theoretical prediction due to additional loss of acoustic energy around the rough surface created by 3D printing^42,43^. A near-perfect absorption peak occurs around 1000 Hz with relative bandwidth of 50% for parameters d1 = 0.5 mm and d2 = 1 mm and has a porosity ${\mathrm{\o }}_{1}$ = 4.91% and ${\mathrm{\o }}_{2}$ = 19.63%, respectively. Here the relative bandwidth is calculated as the ratio of the full width at half the maximum of the absorption coefficient to the resonance frequency. There are two high absorption peaks corresponding to > 0.8 absorption coefficient at 700 Hz and > 0.95 absorption coefficient at 1000 Hz. The small differences in the results of the FEM and theoretical predictions occur due to neglecting the thermal dissipation at the perforation region and considering only viscous energy dissipation.

## Results

### Broadband sound absorption

**Figure 3:**
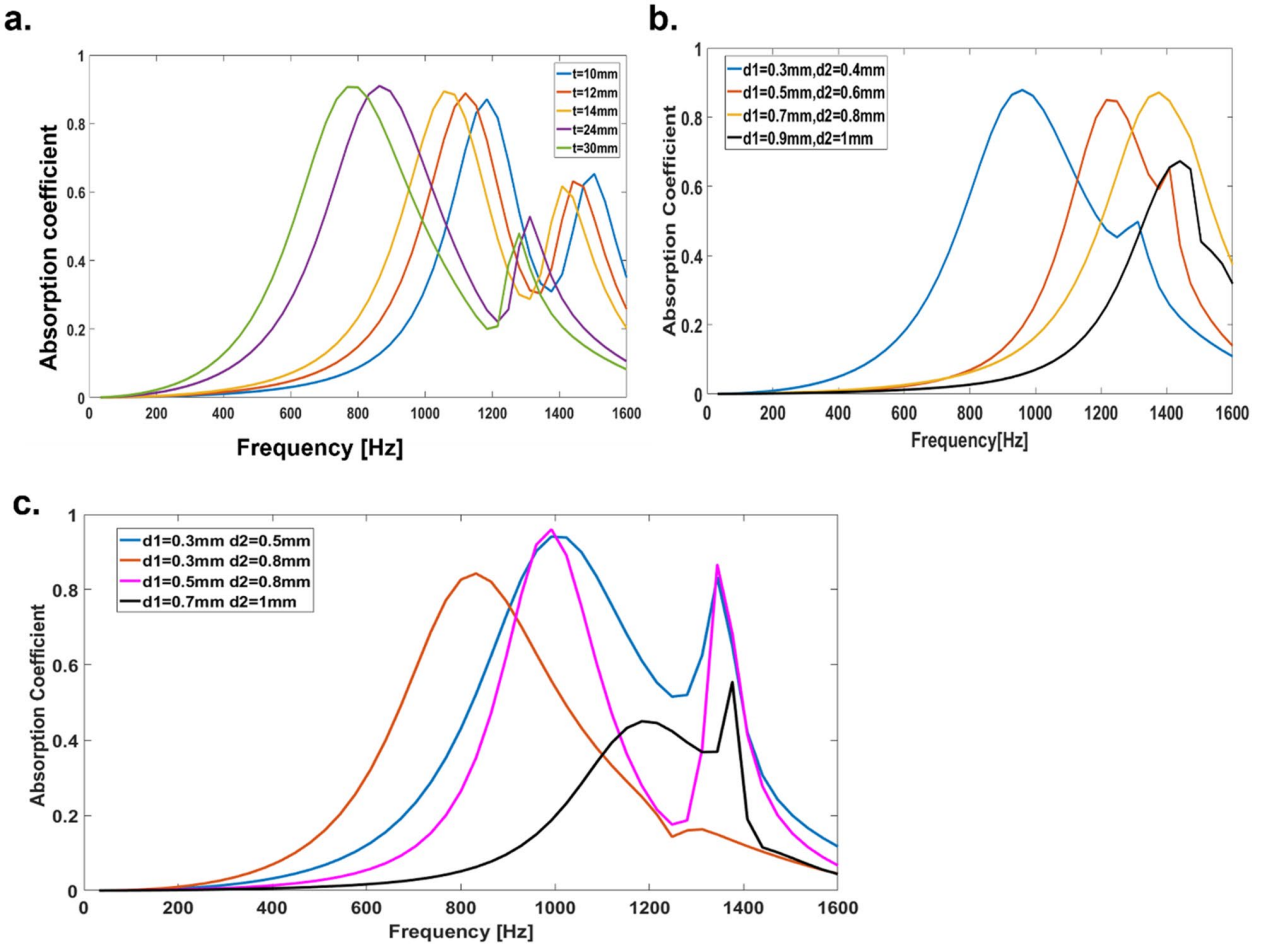
Sound absorption coefficient of CMPP with different acoustic parameters. (**a**) Thickness of the fractals core. (**b,c**) Porosity of CMPP. Courtesy (MATLAB R2016a)^46^.

We start by varying the thickness of the CMPP fractal acoustic metamaterial to achieve varying sound absorption values in the lower frequency range. Given specific values of fixed cross perforation parameter (d1, d2) and porosity of ${\varnothing }_{1}$ and ${\varnothing }_{2}$ Variable fractal core thickness of ‘t’ the sound absorption bandwidth is obtained at a particular frequency, as shown in Fig. 3a.

As the thickness ‘t’ of the fractal core increases, the sound absorption curve gradually shifts from high to low frequency at fixed perforation parameters d1 = 0.5 mm and d2 = 1 mm, respectively. A near-perfect sound absorption peak (96.66%) is obtained at 800 Hz with a relative absorption bandwidth of 50% when t = 30 mm. Similarly, 95.55% sound absorption at 850 Hz, 1150 Hz, 1100 Hz, and 1200 Hz with relative bandwidth (α > 0.5) of 50%, 30%, 29% and 27.6% is obtained as the thickness “t” becomes = 24 mm, 14 mm, 12 mm and 10 mm respectively.

We have also investigated the effect of cross porosity variation on the broadband sound absorption spectra. In the first sample, A1 we have created a geometry corresponding to d1 = 0.3 mm, ${\varnothing }_{1}$ = 7.07% and d2 = 0.4 mm, ${\varnothing }_{2}$ = 12.56%. Similarly, in the second sample A2, the geometrical parameters are changed to d1 = 0.5 mm, ${\varnothing }_{1}$ = 19.63% and d2 = 0.6 mm, ${\varnothing }_{2}$ = 28.26%, in the third sample A3 the parameters are d1 = 0.7 mm, ${\varnothing }_{1}$ = 38.46% and d2 = 0.8 mm, ${\varnothing }_{2}$ = 50.24%, and in the fourth sample, A4, the parameters are changed to d1 = 0.9 mm, ${\varnothing }_{1}$ = 63.59% and d2 = 1 mm, ${\varnothing }_{2}$ = 78.5% respectively. Further other four samples with different porosity combination (A5 to A8) with fractals core have been investigated and their acoustic behavior as shown in Fig. 3c.

The samples are investigated with fixed fractal thickness t = 20 mm. The sample ‘A1’ shows the maximum sound absorption peak (91.2%) to be at 950 Hz with relative bandwidth 58%. Similarly, for the sample A2 and A3 we obtain a similar behavior although as the cross-porosity ratio is increased the resonance frequency is shifted towards the right as shown in Fig. 3b. The sample A4 shows a relatively lower value of 65% absorption at the maximum perforation (details shown in Table 1). The two samples A1 and A5 shows the higher relative bandwidth of sound absorption with maximum sound absorption of 91% and 95%. So, we can tune the novel sound absorbers with a combination of different cross perforation diameters (≤ 1 mm) in each unit cell. For brevity, we have only shown eight combinations here as shown in Fig. 3b,c.

**Table 1:** Fractals CMPPs metamaterials parameters and its acoustic absorption behavior of a unit cell.

S. no.	Samples	Cross porosity	Frequency at maximum absorption coefficient	Relative bandwidth (%)
1	A1	${\varnothing }_{1}$ = 7.07%, ${\varnothing }_{2}$ = 12.56%	@950 Hz (α > 91%)	58
2	A2	${\varnothing }_{1}$ = 19.63%, ${\varnothing }_{2}$ = 28.26%	@1200 Hz (α > 90%)	45
3	A3	${\varnothing }_{1}$ = 38.46%, ${\varnothing }_{2}$ = 50.24%	@1300 Hz (α > 91%)	50
4	A4	${\varnothing }_{1}$ = 63.59%, ${\varnothing }_{2}$ = 78.5%	@1450 (α > 65%)	40
5	A5	${\varnothing }_{1}$ = 7.07%, ${\varnothing }_{2}$ = 19.63%	@1000 Hz (α > 95%)	60
6	A6	${\varnothing }_{1}$ = 7.07%, ${\varnothing }_{2}$ = 50.24%	@800 Hz (α > 81%)	37.5
7	A7	${\varnothing }_{1}$ = 19.63%, ${\varnothing }_{2}$ = 50.24%	@1000 Hz (α > 98%)	25
8	A8	${\varnothing }_{1}$ = 38.46%, ${\varnothing }_{2}$ = 78.5%	@1200 Hz (α > 40%)	0

**Figure 4:**
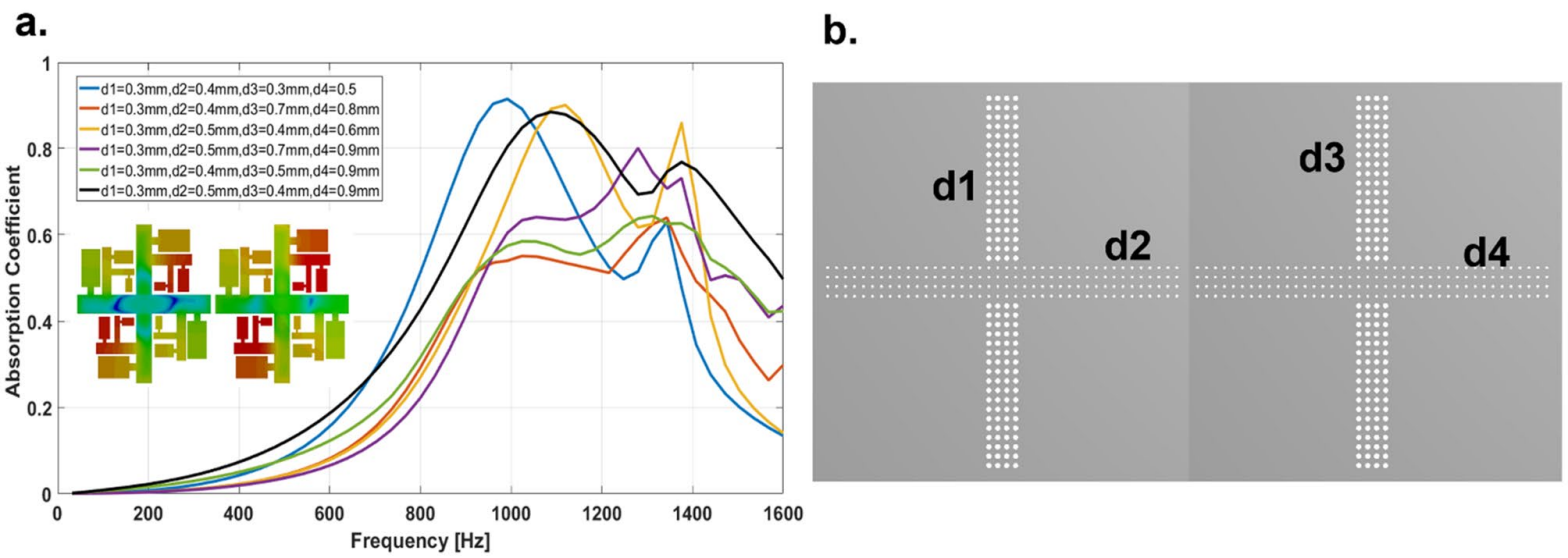
Two-unit cells with different cross perforation. (**a**) Sound absorption coefficient of combined two-unit cells with different cross porosity. (**b**) Top view of the samples of two-unit cells. Courtesy (ANSYS 17.0^45^ and MATLAB R2016a^46^).

In order to expand the relative absorption bandwidth, we have further integrated two-unit cells with different cross porosities into one resonator as shown in Fig. 4b. Unit 1 and unit 2 have the same thickness and fractals cores but with different top CMPP geometries having perforation diameter d1, d2 of unit 1 and d3 and d4 for unit 2. We have also investigated a core containing a pair of unit cells integrated to CMPP and developed six samples S1, S2, S3, S4, S5 and S6 with different geometrical parameters having various combinations of perforation diameters (d1, d2 and d3, d4) and porosity ratios (${\varphi }_{1}$, ${\varphi }_{2}$, ${\varphi }_{3}$ and ${\varphi }_{4})$ (Details in Table 2).

**Table 2:** Fractals CMPPs metamaterials parameters and its acoustic absorption behavior of a two-unit cells.

S. no.	Samples	Cross porosity	Frequency at maximum absorption coefficient	Relative bandwidth
1	S1	${\varphi }_{1}$ = 7.07%, ${\varphi }_{2}$ = 12.56% and ${\varphi }_{3}$ = 7.07% and ${\varphi }_{4}$ = 19.63	1000 Hz (α > 92%)	60%
2	S2	${\varphi }_{1}$ = 7.07%, ${\varphi }_{2}$ = 12.56% and ${\varphi }_{3}$ = 38.46% and ${\varphi }_{4}$ = 50.24%	1300 Hz (α > 60%)	39%
3	S3	${\varphi }_{1}$ = 7.07%, ${\varphi }_{2}$ = 19.63%and ${\varphi }_{3}$ = 12.56% and ${\varphi }_{4}$ = 28.26%	1100 Hz (α > 90%)	47.27%
4	S4	${\varphi }_{1}$ = 7.07%, ${\varphi }_{2}$ = 19.63%and ${\varphi }_{3}$ = 38.46% and ${\varphi }_{4}$ = 63.59%	1250 Hz (α = 80%)	40%
5	S5	${\varphi }_{1}$ = 7.07%, ${\varphi }_{2}$ = 12.56%and ${\varphi }_{3}$ = 19.63% and ${\varphi }_{4}$ = 63.59%	1250 Hz (α > 70%)	41%
6	S6	${\varphi }_{1}$ = 7.07%, ${\varphi }_{2}$ = 19.63%and ${\varphi }_{3}$ = 12.56% and ${\varphi }_{4}$ = 63.59%	1050 Hz (α = 90%)	76% (800–1600 Hz) One octave

Sample S1, shows higher sound absorption coefficient (92%) at 1000 Hz and 60% relative bandwidth, sample S2 has a 76% absorption coefficient with approximately one-octave sound absorption bandwidth, t thickness 20 mm as shown in Fig. 4a. All the samples show broader relative sound absorption bandwidth within the range 39–76% and the approach shown here demonstrates the ability to customize the sound absorption bandwidth as per requirements by careful consideration of the correct combination of the core with various CMPP geometries.

**Figure 5:**
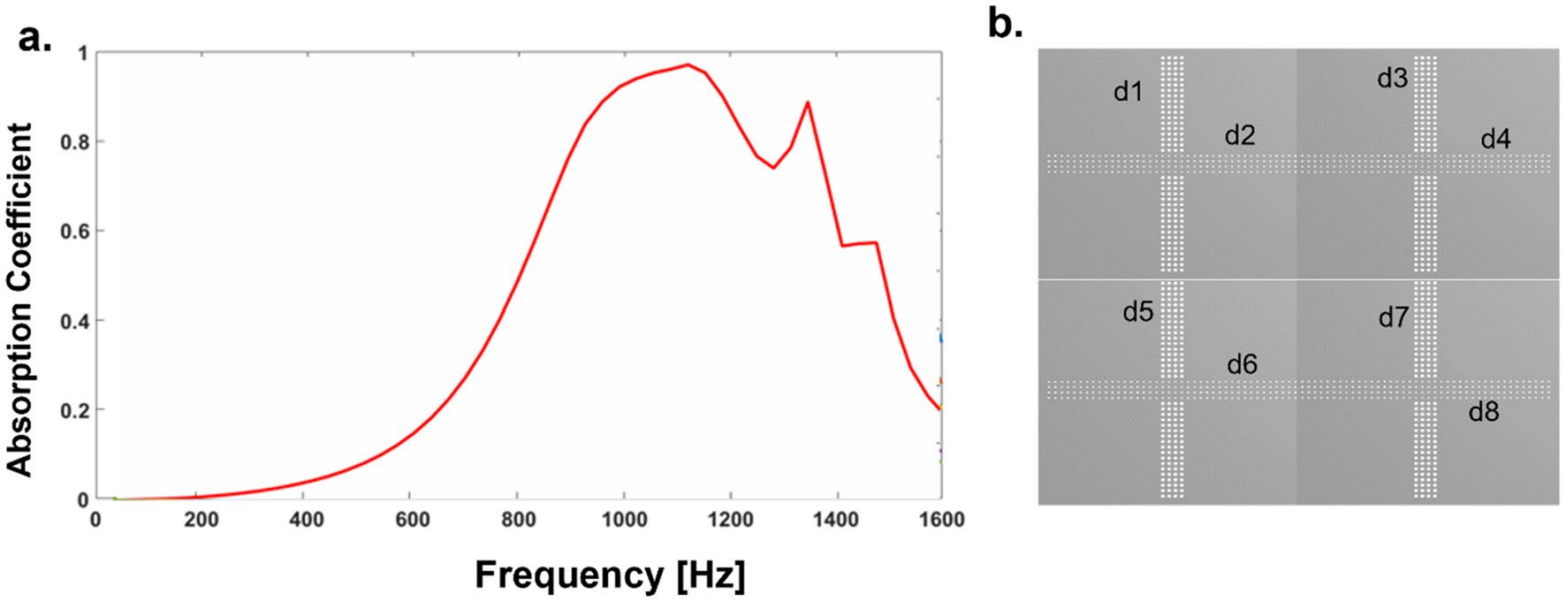
Four-unit cells with different cross perforation. (**a**) Sound absorption coefficient of combined four-unit cells with different cross porosity. (**b**) Top view of the samples of four-unit cells. Courtesy (ANSYS 17.0^45^ and MATLAB R2016a^46^).

We have further integrated 4 different unit cells reported in Fig. 5b, to get a broader sound absorption response from these geometries. The porosity of the cross MPPs is optimized numerically to get suitable combinations to achieve a maximized sound absorption bandwidth. The perforation dimensions are d1 = 0.3 mm, d2 = 0.4 mm, d3 = 0.5 mm, d4 = 0.6 mm, d5 = 0.7 mm, d6 = 0.8 mm, d7 = 0.9 mm and d8 = 1 mm.

As we can see from the Fig. 5a that the sound absorption bandwidth from 800 to 1400 Hz demonstrates an absorption coefficient of greater than 80% and the average relative bandwidth of 61%.

## Discussion

Effective and efficient Attenuation of noise requires limited thickness, light weightiness and perfect sound absorption performance in broadband frequencies, especially in the lower frequency range. We have proposed a novel class of cross micro-perforated hybrid acoustic metamaterial with Helmholtz fractal cores that possess outstanding sound absorption over broadband low frequency range with excellent tunability. Using electrical analogy methods the equivalent impedance to sound propagation within the fractal core is evaluated in combination with the classical improved Maa Model^25^, for the CMPPs. We have developed a theoretical approach to calculate the equivalent sound absorption coefficient for a bunch of geometric combinations. This theory is then validated through a numerical approach (FEM) as well as experimentally. The results show that novel sound absorbers 20 mm thickness can achieve near perfect absorption around 1000 Hz, with a broadband absorption bandwidth. Approximately 1 octave band sound absorption coefficient > 0.5 have been achieved with single unit cell and more than 0.8 has been achieved within the frequency range 600–1100 Hz. Maximum relative sound absorption bandwidth of 76% has been achieved with an integrated two unit cell configuration and 61% with 4 unit cell combination. The sound absorption coefficient has been increased by integrating the unit cells.

## Methods

### Numerical simulations

**Figure 6:**
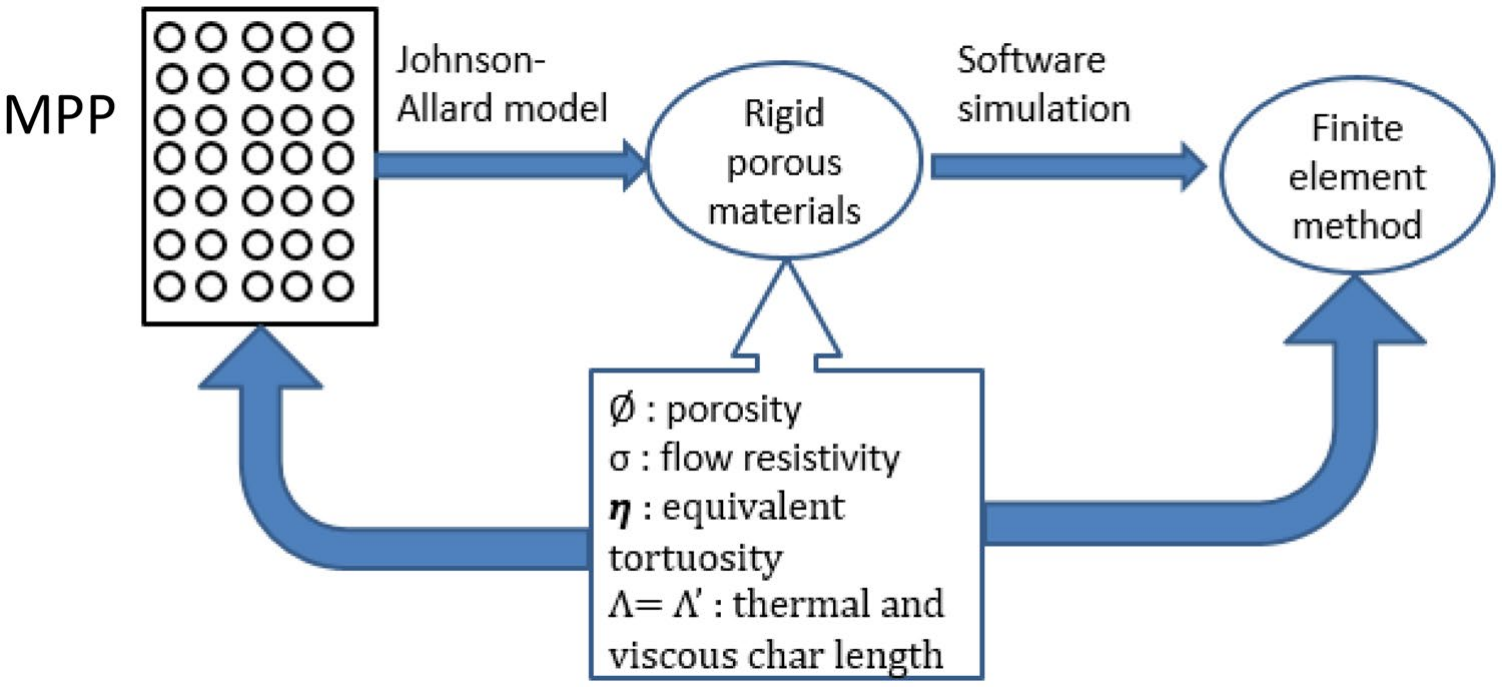
Equivalent fluid model conversion of MPP to rigid porous materials using the parameters of Ø, σ, η, Λ and Λʹ.

**Figure 7:**
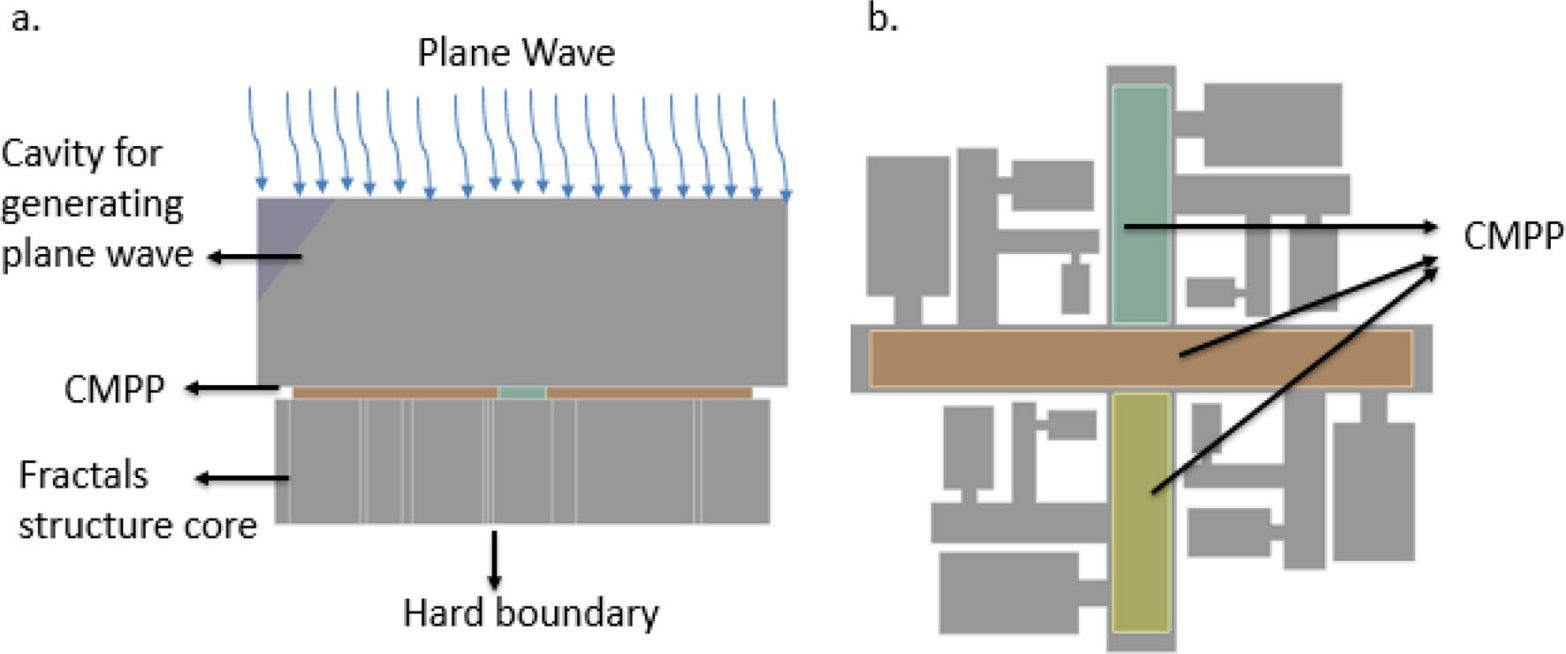
FEM simulation setup to analyze the sound absorption coefficient. (**a**) Simulation setup. (**b**) Equivalent fluid model of proposed CMPP fractals acoustic metamaterials. Courtesy (ANSYS 17.0^45^).

The sound absorption coefficient of CMPP is carried out using ANSYS 17.0^44^, with its acoustic module. We have approached the problem by first converting the MPPs into rigid porous materials and used equivalent fluid model in numerical analysis FEM simulation to obtain the final estimate (Fig. 6). The equivalent fluid cad model of CMPPs as shown in Fig. 7b.

The equivalent fluid model is defined with the parameters that can be calculated by the Eqs. (9) to (11). These calculated values have been used in FEM simulations as shown in Fig. 7 below.

9 $$\Lambda = {\Lambda }^{^{\prime}}=\frac{d}{2},$$

10 $$\sigma =\frac{32\tau }{\varnothing {d}^{2}},$$

where $\tau$ is dynamic viscosity and $d$ is the diameter of the perforation

11 $$\eta =1+\frac{2\times 0.48\sqrt{\pi {r}^{2}}(1-1.14\sqrt{\varnothing })}{t},$$

where $r$ is the radius of the perforation and t is the thickness of the CMPP.

The 3D model of CMPP acoustic metamaterials shown in Fig. 7a created in the DesignModeler of ANSYS 17.0^45^. A plane wave with unit amplitude is applied normally and hard boundary conditions is applied on all the walls of the interface between air and the surface at the subsurface levels of the structure.

## Supplementary Information

Supplementary Information.

## Data Availability

The datasets generated during and/or analyzed during the current study are available from the corresponding author on reasonable request.

## Code availability

The FEM simulations were performed using ANSYS 17 (Academic)^45^ and for plotting of all the graph, and theoretical coding, used MATLAB (R2016a)^46^ (License No. 40765629). The code is available from (bhattacs@iitk.ac.in) on reasonable request.
