# Supplementary material for: Hybrid fractal acoustic metamaterials for low-frequency sound absorber based on cross mixed micro-perforated panel mounted over the fractals structure cavity
Source: Sci Rep. 2022 Nov 28;12:20444. doi: 10.1038/s41598-022-24621-8 (PMC9705723; doi:10.1038/s41598-022-24621-8)
Supplement: Supplementary file 1 — Supplementary Information. [file 41598_2022_24621_MOESM1_ESM.docx]

**Supplementary materials**

**Hybrid fractal acoustic metamaterials for low-frequency sound absorber based on cross mixed micro-perforated panel mounted over the fractals structure cavity**

Sanjeet Kumar Singh^1^, Om Prakash^3^ and Shantanu Bhattacharya*^1,2^

^1^Department of design, Indian Institute of Technology Kanpur, India.

^2^Microsystem Fabrication Laboratory, Department of Mechanical Engineering, Indian Institute of Technology Kanpur, 208016, Uttar Pradesh India.

^3^Boeing International Corporation India Private Limited, RMZ Infinity, Tower D, 5th Floor, Old Madras Road, Bengaluru 560001, Karnataka, India

*Corresponding Author email: [bhattacs@iitk.ac.in](mailto:bhattacs@iitk.ac.in)

**Note S1 : Impedance of the fractals cavity**

We have first calculated the equivalent impedance of one unit cell of the cavity through an electrical analogy^1^ developed as shown in fig 1 (c)


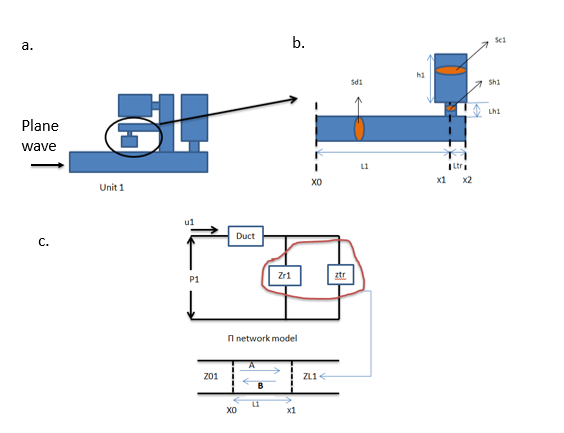


**Figure 1.** (a) One representative branch of the fractal structure core metamaterial which consist of four equal branches. (b) Representative side branch of Helmholtz resonator structure in the fractals. (c) Electrical analogy using network model and their corresponding equivalent impedance.

Zc1=$\frac{\rho.c}{sc1}$ (1)

$zr1$and $ztr1$ are parallelly connected enabling us to use parallel circuit connection model as below (electrical analogy).

Zx1=$\frac{zr1.ztr1}{zr1+ztr1}$ (2)

$zr1$=-j.zc1.cot(k.h1)+Z_h_ (3)

$ztr1=-j.z_{d1}.cot(k.Ltr$) (4)

Where $z_{d1}=\rho_{0}c_{0}/sd1$ characteristics impedance

where Zh=($\frac{\rho.c}{sh1})(0.0072+j.k.(Lh1+0.75)$ (5)

Using equations 1 ,2,3,4,5 we can calculate the impedance of the first side branch Helmholtz resonator ^2^

Z01=$z_{d1}\frac{\left( \frac{zx1}{zd1} \right)+jtan(k.L1)}{1+j.\left( \frac{zx1}{zd1} \right)tan(k.L1)}$ (6)


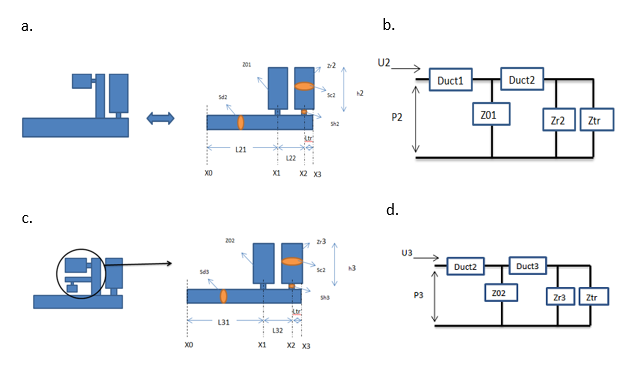


**Figure 2**. (a) Second iteration of fractals geometry and its equivalent side branch resonators model. (b) Electrical analogy or ᴨ network model of second iteration. (c) Third iteration of fractals geometry and its equivalent side branch resonators model. (d) cross-ponding Electrical analogy or ᴨ network model of the third iteration

We now calculate the equivalent impedance of the second iteration as shown in fig 2 (a, b), with the help of series and parallel electrical circuit analogy and repeat the same procedure as above

Zx2 = $\frac{Zr2 . Ztr2}{Zr2+Ztr2}$ (7)

Zx1 = Zd2 $\frac{\left( \frac{Zx2}{Zd2} \right)+j tan(kL22)}{1+j \left( \frac{Zx2}{Zd2} \right)tan (kL22)}$ (8)

Where$ztr2=-j.z_{d2}.cot(k.Ltr$) and $z_{d2}=\rho_{0}c_{0}/sd2$

Z_eq_ =[ (1/Z01) +(1/Zx1)]^-1^

Z02 = Zd2 $\frac{\left( \frac{Zeq}{Zd2} \right)+j tan(kL21)}{1+j\left( \frac{Zeq}{Zd2} \right)tan(kL21)}$ (9)

Similarly we use the same procedure for the third iteration as shown in fig. 2 (c, d)

Zx2 = $\frac{Zr3 . Ztr3}{Zr3+Ztr3}$ (10)

Zx1 = Zd3 $\frac{\left( \frac{Zx2}{Zd3} \right)+j tan(kL32)}{1+j \left( \frac{Zx2}{Zd3} \right)tan (kL32)}$ (11)

Where$ztr3=-j.z_{d3}.cot(k.Ltr$) and $z_{d3}=\rho_{0}c_{0}/sd3$

Z_eq_ =[ (1/Z02) +(1/Zx1)]^-1^

Equivalent impedance of the first branch of the fractal acoustic metamaterials as

Z03 = Zd2 $\frac{\left( \frac{Zeq}{Zd3} \right)+j tan(kL31)}{1+j\left( \frac{Zeq}{Zd3} \right)tan(kL31)}$ (12)

There are 4 identical cavity placed parallelly so the total impedance of the cavity

Z fc = Z03/4 (13)

**Note S2: Measurement of sound absorption (experimental apparatus)**

A sample is further built using 3D printer with a diameter of 100mm as shown in figure 4(a). The material used for fabrication of the sample is polylactic acid (PLA). The speed of sound and the density of the used PLA are c= 1200 m/s and ρ = 2700 kg/m3 respectively used for estimating the sound wave particle velocity flowing inside the structure as shown in Figure 4(b). The absorption coefficient of the fabricated specimen within a range of 400 -1600 Hz acoustic frequencies is shown in figure 4(c), which is measured on a B&k type-4206T impedance tube system. The acoustic absorption coefficient is measured following the ASTM E1050–12 standard.4 The following parts are used to make the impedance setup: (a)An impedance tube (inner size is of 10x10cm2 ), (b) two microphones (1/4-in.-diameter) (Labeled M1 and M2), (c) a measuring module “Acoustic Material Testing” which is used to measure the absorption coefficient. The thickness of the waveguide wall is treated as 6mm. Since the rear of the wall is a hard boundary condition, we can assume that there is no sound transmission through the wall. By placing a structured material block inside the tube, the absorption spectra can be measured for the corresponding meta-surface of this material block. Figure 3 below shows the complete experimental setup used for measurement of the absorption coefficients. All experiments have been performed at laboratory conditions with ambient temperature and relative humidity as 25 °C and 50%, respectively. The speed of sound is assumed as 343.2 m/s and the density of air as 1.2 kg/m3. The Impedance tube has a horizontal columnlike structure with acoustic source (speaker) at one end and the microphone holders embedded along the tube length for conducting the measurements. The other end of the impedance tube has been provided with rigid termination. The loudspeaker is connected to a signal generator for producing a random signal and thus generates planar acoustic waves to propagate through the tube. Both microphones are placed in holders provided along the length of tube. Outputs of these two microphones are connected to a data-acquisition system which has a computer interface. Transfer function method has been used to measure absorption coefficient of the samples. This method separates the incident and reflected sound pressure through the measured transfer function between two microphone locations, and then calculates acoustic absorption coefficient of the sample. The measurement procedure is as per the standard ASTM E1050-12


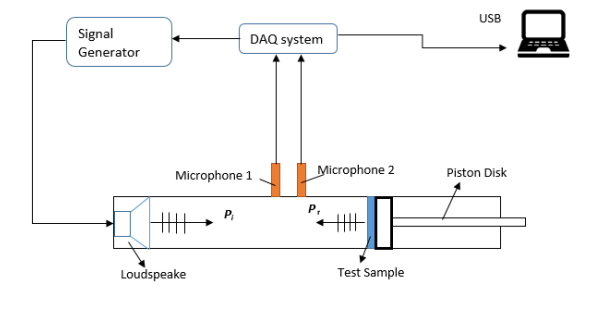
Figure 3. Schematic of the sound absorption experiment using an impedance tube and two microphone technique

**Note S3. Dimensions of the base unit and Response surface methodology optimization**


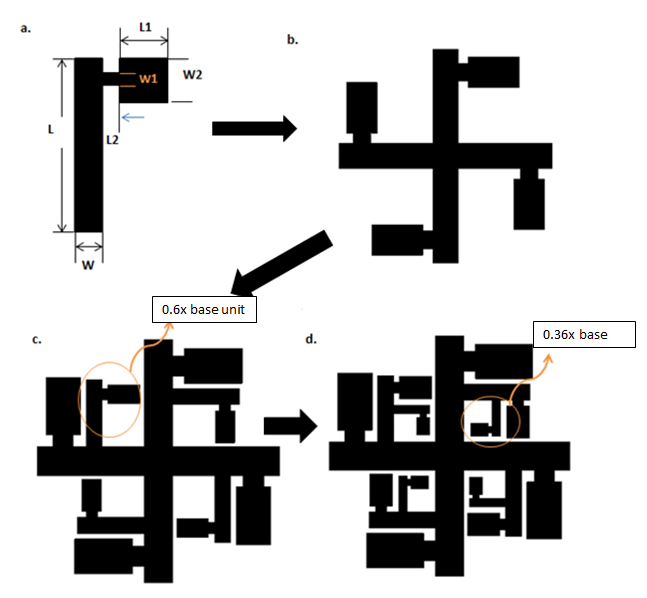


Figure 4. Base unit of the designed meta-metamaterials

We optimized the proposed structure at the targeted frequency of 1000Hz and with a single hole at the center^1^. Now this optimized structure is combined with the cross-perforation plate (CMPP) , constructed using two different hole diameters “d1” and “d2”,to get broadband sound absorption.

Numerical and graphical optimization algorithm is performed, using design Expert 8.0

To maximize || *sound absorption coefficient (*$\alpha\left( f \right)=$-1105.72 +11979.01* A - 2661.78* B+272.75* C+0.6820* D-0.2008* A * B+11978.56* A * C+0.7707*A * D-2662.29* B * C-0.0236* B * D+0.3695* C * D+7985.91* A2+0.2713* B2 +2436.62* C2 + 0.0236*D2+0.6126*A*B*D -0.3315*A2*B+7985.89*A2*C+0.0348*A2*D-0.7647A*B2-0.3227*A*D2-0.6108*B^2^*D+0.5445 *A3-0.2662*B3+

+1057.72*C3-0.0005*D^3^+0.2589* B4

Subjected to:

10 ≤$d_{p}$ ≤ 14

2 ≤ $\left( \frac{w2}{w1} \right)$ ≤ 6

6.67≤ ${l_{1}}/{l_{2}}$ ≤ 20

6 ≤ $t_{c}$ ≤ 9

where

A=dp (diameter of the hole)

B=w2/w1

C=$\frac{l1}{l2}$

D=tc (thickness of the structure)

| **Method** | $\boldsymbol{d}_{\boldsymbol{p}}$ | $\frac{w2}{w1}$ | ${\boldsymbol{l}_{\boldsymbol{1}}}/{\boldsymbol{l}_{\boldsymbol{2}}}$ | $\boldsymbol{t}_{\boldsymbol{c}}$ | $\boldsymbol{\alpha(1000)}$ |
| --- | --- | --- | --- | --- | --- |
| Design Expert numerical optimization | 11.68 | 2 | 20 | 8.5 | 0.99 |
| ANSYS 17.0 Simulation | 12 | 2.4 | 20 | 8 | 0.92 |

**References**

1. Singh, S. K., Prakash, O. & Bhattacharya, S. Novel fractal acoustic metamaterials ( FAM ) for multiple narrow band near perfect absorption. Journal of applied physics, Accepted, June 27^th^, 2022, In print

2. Seo, S.-H. & Kim, Y.-H. Silencer design by using array resonators for low-frequency band noise reduction. *J. Acoust. Soc. Am.* **118**, 2332–2338 (2005).
